# Effect of carnosine, aminoguanidine, and aspirin drops on the prevention of cataracts in diabetic rats

**Published:** 2008-12-11

**Authors:** Hong Yan, Yong Guo, Jie Zhang, Zhenghua Ding, Wenjing Ha, J.J. Harding

**Affiliations:** 1 Department of Ophthalmology, Tangdu Hospital, Fourth Military Medical University, Xi’an, China; 2 Nuffield Laboratory of Ophthalmology, University of Oxford, Walton Street, Oxford, UK

## Abstract

**Purpose:**

To investigate the effect of carnosine (CA), aminoguanidine (AG), and aspirin (ASA) drops, all inhibitors of glycation, on the development of diabetic cataract in rat.

**Methods:**

Rats were made diabetic with streptozotocin, and based on the level of plasma glucose, they were assigned as non-diabetic rats (<14 mmol/l plasma glucose) and diabetic rats (>14 mmol/l plasma glucose). Animals in the treated groups received CA, AG, and ASA as drops to the left eyes starting from the day of streptozotocin injection. Progression of lens opacification was recorded using the slit lamp at regular time intervals. All the rats were killed after the week 13, and the levels of advanced glycation end products (AGE), glutathione reductase (GR), catalase (CAT), and glutathione (GSH) were determined.

**Results:**

Lens opacification progressed in a biphasic manner in the diabetic rats, an initial slow increase during the first eight weeks of diabetes followed by a steep increase in the next five weeks. Carnosine treatment delayed the progression of cataracts in diabetic rats, and the delay was statistically significant on the fourth week of diabetes (p<0.05, when compared with untreated moderately diabetic rats). A decrease in the antioxidant enzymes of CAT and the level of GSH was found in the lens of the untreated diabetic rats at 13 weeks after injection. Some protection was provided in the treated eyes. The level of glycation in the untreated diabetic rats was significantly higher than that in the normal rats (p<0.001). After treatment with CA, AG, and ASA, those diabetic rats had a lower level of glycated lens protein compared to the untreated diabetic rats (p<0.001).

**Conclusions:**

These results thus suggest that the effect of CA, AG, and ASA is indeed inhibition of the formation of AGEs. However, the effect of CA, AG, and ASA is overwhelmed by the excessive accumulation of AGEs in the severely diabetic rats. CA compared with AG and ASA treatment can delay the progression of lens opacification in the diabetic rats only during the earlier stages. It also protects against the inactivation of enzymes.

## Introduction

Cataract is a leading cause for blindness. Worldwide 17 million people are blind because of cataract formation, and the problem will grow in parallel with aging of the population [1,2]. Surgical treatment with phacoemulsification and intraocular lens implantation remains the only proven treatment. This, however, is associated with significant cost and is not readily available especially in the developing countries where the prevalence of cataract is the highest. The serious complications as a result of cataract surgery are inevitable and expensive. Thus, risk and cost factors drive the study of pharmaceutical approaches to the maintenance of lens transparency, and it is therefore a highly desirable alternative.

Cataracts are aggregates that result from dysfunctional protein interactions leading to increased lens opacity [1]. The cause of protein aggregation and hence lens opacity remains poorly understood. Posttranslational modifications (PTM) of lens crystallins, consequent to aging or diseases such as diabetes, may result in conformational changes and aggregation of these proteins and lead to lens opacification and cataract formation. Chemical modifications of lens proteins and enzymes by reactions such as glycation play an important and pivotal role.

Glycation, the non-enzymic reaction of sugars with protein, occurs normally but to an increased extent during aging and diabetic complications [3]. Glycation and other non-enzymic posttranslational modifications of proteins have been implicated in the complications of diabetes and other conditions. It can lead to protein cross-linking and further aggregates, which contribute to the pathogenesis of conformational diseases including cataract and complications of diabetes [3]. Glycation leads to protein cross-linking, which may act as a nucleation site that causes further aggregation. Glycation and especially advanced glycation producing cross-linking cause a decrease in chaperone function of α-crystallin and the inactivation of enzymes [4-7]. Advanced glycation end products (AGEs) induced by hyperglycemia contribute to cataract formation. Medical treatment of cataract through the prevention of these modifications and its consequences is therefore a highly desired alternative [8,9].

Carnosine (CA), a naturally occurring dipeptide (beta-alanyl-L-histidine), is found predominantly in long-lived tissues including the brain, innervated muscle, and the lens in surprisingly high amounts (up to 20 mM in human muscle) [10]. It appears to possess antiglycating, antioxidant, and free-radical scavenging activity and could be useful in any degenerative condition where posttranslational modification plays a role [11-13]. It was discovered by the Russian scholars, Gulewitsch and Amiradzibi, in 1900. Other Russian scholars, namely Severin and Boldyrev [14], made great contributions to research on the biological effects and medical application of CA. CA can inhibit cross-linking of proteins [10,12,15] and protects against the inactivation of enzymes induced by glycation, oxidation, and a steroid [16-18]. In addition, CA can modulate the reactivity of the glycated protein toward α-crystallin [19], directly reacts with sugars [10], and inhibits modifications and the decreased molecular chaperone activity of lens α-crystallin induced by glycation [7]. Protein cross-linking is a consequence of glycation that induces formation of carbonyl (CO) groups. CA can react with protein CO groups and thereby modulate their deleterious interaction with other polypeptides [19]. The potential biological and therapeutic significance of CA against human age-related cataract and canine cataract has been reported, indicating that CA has the potential of being a drug to prevent and treat cataract [14,20-22].

Aminoguanidine (AG) as an antiglycation drug can inhibit a variety of diabetic complications by trapping the reactive carbonyls and preventing the formation of AGEs [23-26]. Diabetics have increased glucosamine levels. It is possible that the glycation of the lens structural proteins by glucosamine induces conformational changes in the lens that contribute to cataract formation [27].

AG can delay the development of diabetic cataract [26,28,29], indicating that cataract formation in diabetes involves glycation. The reduced formation of acellular collapsed capillary strands by AG suggests a potential role for glycation in vascular damage [29]. AG can delay cataract development in UPL rats (a newly developed hereditary cataract model) possibly by inhibiting the rise in Ca^2+^ levels [30]. Aspirin (ASA) and ASA-like analgesics have been studied in a variety of model systems including diabetic rats [31]. A variety of laboratory and epidemiological evidence supports the benefits of ASA-like drugs, but there has been no clinical trial specifically in patients with cataract [32-34].

However, none of the clinical trials of other drug therapies showed any convincing anti-cataract effect of the compounds or mixtures tested. Therefore, a successful anti-cataract drug still remains to be developed. We previously used the streptozotocin (STZ)-induced cataract model in rats successfully to assess ASA, paracetamol, ibuprofen [31], N-acetylcysteine, and glutathione ethyl ester eye drops [35] as anti-cataract drugs. In this study, we investigate CA, AG, and ASA as putative anti-cataract drugs in parallel to assess which can delay diabetic cataract in rats. We applied the drugs topically as this minimizes the possibility of side effects. The three eye drops were used for the first time in a streptozotocin-induced cataract study.

## Methods

### Materials

Streptozotocin, CA, AG, ASA, and 5-hydroxymethylfurfural (5-HMF) were purchased from Sigma Chemical Co (Beijing, China). Protein and enzyme quantification kits were obtained from Jiancheng Biology Company (Nanjing, China). Glucotrend 2 was from Roche Diagnostics Limited Company (Xi’an, China). Test-Tape was from Zhujiang Biological and Chemical Reagents Company (Guangzhou, China). All other chemicals and solvents were of analytical grade and were obtained from local companies.

### Animal model

The experiments, which lasted over 14 weeks, were performed using Sprague-Dawley male rats (Laboratory Animal Research Centre, Fourth Military Medical University, Xi’an, China), which were housed in individual polypropylene breeding cages under a day/night cycle of 12 h at room temperature (20 °C−25 °C). Animal care and protocols were in accordance with and approved by the Institutional Animals Ethics Committee and conformed to the ARVO Statement for the Use of Animals in Ophthalmic and Vision Research.

All the lenses were examined at the slit lamp before the induction of diabetes, and those with any defect in the lens or cornea were rejected. The rats were weighed, and seven groups were established with a comparable weight distribution (ranging from 112.0±19.3 g to 143.0±16.2 g; p>0.05).

The rats in diabetic groups were injected intraperitoneally, avoiding the intestine, with streptozotocin (65 mg/kg bodyweight) dissolved in 20 mM sodium citrate buffer, pH 4.5 (10 mg streptozotocin per 1 ml citrate buffer). The streptozotocin solution was sterilized through a 0.22 μm Millipore filter (Oxford Immunotec Ltd., Oxford, UK) into a sterilized container kept on ice and used within 10 min of dissolving. The non-diabetic rats (group I, n=5) were injected with sterilized buffer alone. After three days, the blood of each rat was tested for glucose with Glucotrend 2 (Roche Company, Basel, Switzerland). The urine of each rat was tested for glucose with Test-Tape at the same time. Any streptozotocin-injected rats that had no detectable glucose in the urine were rejected. Fasting blood glucose was then measured in all rats to confirm that the streptozotocin-injected rats were diabetic (more than 14 mM) and that the control rats were not. In this study, diabetes was assessed by the measurement of sugar both in blood and in urine at the same time. We found that measuring urine sugar is simple, less expensive, and highly reliable. Although the measurement of urine sugar is simple, the result is not precise. The blood glucose of rats varies in 24 hours. Therefore, the use of simultaneous measurement of urine sugar and blood glucose is the better way compared to measurement of urine sugar or blood glucose alone.

The diabetic rats (n=59) were then divided randomly into groups II–VII and received 5 mg/ml CA drops (group II; n=9), 10 mg/ml CA drops (group III; n=9), 0.5 mg/ml ASA drops (group IV; n=9), 1 mg/ml AG drops (group V; n=9), and 2 mg/ml AG drops (group VI; n=9), all in 25 mM sodium phosphate buffer (pH 7.4). The untreated diabetic group constituted group VII (n=14). The rats were initially given no treatment until it was certain that those rats in groups II–VII were diabetic. Then, eye drops consisting of 25 mM sodium phosphate buffer (pH 7.4) with the relevant drugs dissolved in it were administered twice daily to the left eyes of the treated groups, and the untreated diabetic group (group VII) received the buffer solution alone. The drug solutions were made fresh daily. The rats were fed with standard chow ad libitum. Only 22% (14/64) of diabetic rats and none of the controls suffered unscheduled deaths. The deaths were mostly toward the end of the experiment.

### Monitoring rat corneal staining

To evaluate the safety of eye drops, the extent of the corneal staining was observed. After anesthetizing the rats using ether vapor, 2% fluorescein sodium solution was dropped into the rat eyes, and rat corneal staining was monitored using a slit lamp (Haag-Streit BQ900, Koeniz, Switzerland). The slit width was 0.3 mm, and light angle was 45°. Magnification was 50X. The photographs of rat corneal staining were taken monthly with a BQ900 slit lamp camera (Haag-Streit BQ900). The extent of corneal staining was graded according to the method described by Wander et al. [36] with minor modifications: Grade 0, no staining; grade 1, up to a quarter of the cornea stained; grade 2, a quarter to a half of the cornea stained; grade 3, more than half of the cornea stained.

### Observation of cataract

**Figure 1:**
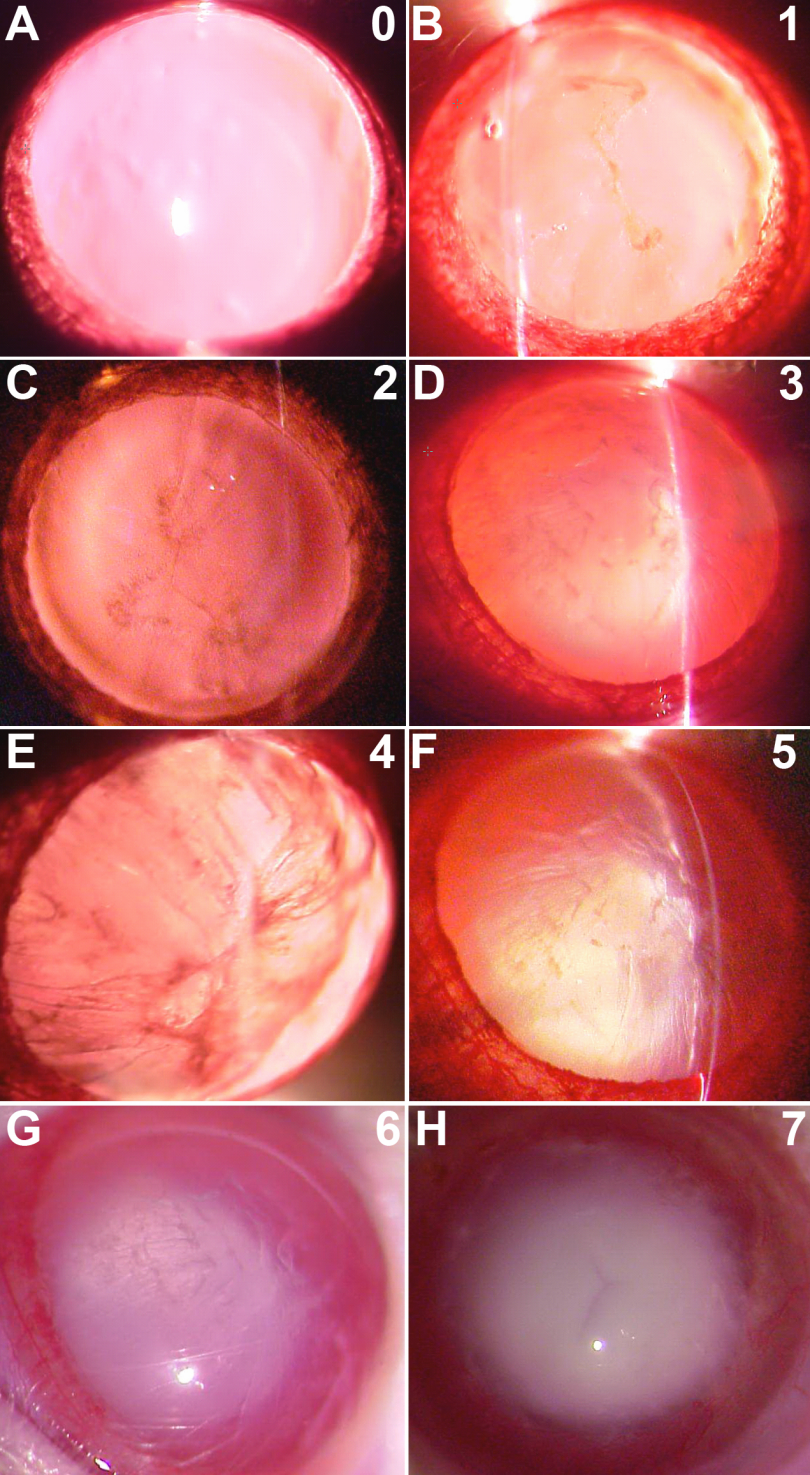
Progression of cataract in the left lenses of untreated diabetic rats, group VII, assessed at the slit lamp. The grades are as follows: grade 0, clear; grade 1, clear nuclear with wide sutures; grade 2, slight dense nuclear with opacities radiating from sutures; grade 3, dense nuclear without clefts; grade 4, dense nuclear with clefts; grade 5, nuclear cataract with clefts; grade 6, nuclear cataract with dense radial opacities; and grade 7, nuclear cataract with whole lens opacities.

Progression of cataract formation in both lenses of all rats was followed by slit lamp observation using a Haag-Streit BQ 900 model and recorded by photography. The pupils were dilated with a drop of tropicamide, and after at least 5 min, the rats were anaesthetized by ether inhalation. Grading of lens opacification was performed according to the Oxford system [31], and the initiation, progression, and maturation of lenticular opacity was graded into seven stages as follows: grade 0, clear; grade 1, clear nucleus with wide sutures; grade 2, slight dense nucleus with opacities radiating from sutures; grade 3, dense nucleus without clefts; grade 4, dense nucleus with clefts; grade 5, nuclear cataract with clefts; grade 6, nuclear cataract with dense radial opacities; grade 7, nuclear cataract with whole lens opacities (Figure 1). The stage of cataract was scored according to the classification described above.

### Lens preparation

The rats were sacrificed, and the lenses were removed for measurement of the level of glutathione (GSH) and glycated protein and of the activity of glutathione reductase (GR) and catalase (CAT). The eyes were removed, and the lenses were dissected out, placed into pre-weighed Eppendorf tubes, and frozen until they were analyzed. Left and right lenses were assayed in the same way.

### Free, reduced glutathione in the lens

The measurement of free, reduced GSH was performed using the 5,5′-dithiobis-[2-nitrobenzoic acid] (DTNB) method [37]. The right and left lenses were defrosted within six days of dissection and freezing and ground in 1 ml 10% trichloroacetic acid (TCA) then left for 20 h before centrifugation at 11,500x g at room temperature for 20 min. The supernatant was removed into a pre-weighed tube. The precipitate was washed twice with 10% TCA and centrifuged for 20 min and the supernatants combined. The protein precipitate was retained for measurement of the glycated proteins. Two lots of 0.5 ml supernatant from each tube were made up to 1 ml by adding 10% TCA and 2 ml of 1 M Tris, pH 9.0 followed by 50 μl DTNB (dissolved in ethanol at a concentration of 3.965 mg/ml) and mixed well. Standards were made using different volumes of 0.1 mM reduced GSH in 10% TCA and making up to 1 ml with 10% TCA. Reaction blanks consisted of 1.0 ml 10% TCA, Tris, and DTNB. The reaction solution duplicates after shaking when left to stand for 5 min at room temperature, and the absorbance was read at 412 nm. The concentration of GSH was read from a standard graph.

### Glycation of lens proteins

Determination of glycation of lens proteins was based on the method described by Blakytny and Harding [4]. Protein that precipitated during the determination of GSH in both the right and left lens was suspended in 1 ml diethyl ether to remove TCA and left for 10 min at room temperature before centrifugation for 5 min at 11,500x g. The supernatant was removed and the pellet washed twice more with diethyl ether (1 ml and 0.5 ml). The pellet was dried in a fume cupboard to remove any remaining diethyl ether. Then, upon boiling glycated protein in the presence of a weak acid, 5-hydroxymethylfurfural (5-HMF) was released into solution. Any solubilized protein was then precipitated out of solution. Thiobarbituric acid forms an adduct with 5-HMF that absorbs at 443 nm.

Dried protein pellets (25 mg) were weighed out into twist-top vials to which 1 ml of 1 M oxalic acid was added. Standards consisted of 5-HMF in 1 ml of 1 M oxalic acid and the blanks being 1 ml of 1 M oxalic acid alone. The vials were kept at 100 °C for 2 h and then cooled to room temperature before removal of 0.15 ml for protein determination. The remaining solution was transferred to centrifuge tubes to which 1 ml of 40% TCA was added and cooled in an ice bath for 10 min to allow protein precipitation. The tubes were centrifuged for 15 min at 3,500x g at room temperature, and 2×0.8 ml of the supernatant from each tube was placed in Eppendorf tubes for further centrifugation at 11,500x g for 5 min at room temperature. The supernatant from each of the tubes (0.75 ml) was incubated with 0.25 ml of 0.05 M thiobarbituric acid (TBA), adjusted to pH 6.0 with 1 M sodium hydroxide at 40 °C for 40 min, and then allowed to cool at room temperature for 15 min. The absorbance was read at 443 nm against a blank, and glycation was determined from a standard graph using 5-HMF. After boiling in oxalic acid, 0.15 ml was removed (see above) and diluted 100 fold. Then, two 1 ml aliquots of this diluted protein were used for protein assay.

### Assay of glutathione reductase and catalase activity

Protein concentration was determined by Lowry's method using a protein assay kit from Jiancheng Company. Glutathione reductase (GR) and catalase (CAT) was determined using an assay kit from Jiancheng Company. GR activity was measured according to the procedure in which oxidized glutathione (GSSG) was reduced to GSH, which was catalyzed by GR with NADPH as the cofactor. The decrease in the optical density at 340 nm was recorded at 25 °C for 2 min. The units of enzymatic activity were calculated using an extinction coefficient of 6.22 mM•cm^−1^ for NADPH. One unit was equivalent to the oxidation of 1 mmol of NADPH per min. CAT activity in the lens was assayed with hydrogen peroxide as the substrate through the use of a method based on the direct measurement of H_2_O_2_ decomposition. The final volume of each enzyme assay was 3 ml of substrate and 20 μl of the supernatant of lens homogenate. The assay was performed at 25 °C and at 240 nm. Enzyme activity was expressed as units per gram of protein, and one unit of CAT activity represented 1 mmol H_2_O_2_ decomposed per min.

### Data analysis

Unless otherwise stated, differences were assessed using Student’s *t* test. Analysis of cataract grades in different rat groups was analyzed on individual lenses using a Wilcoxon Signed Ranks Test.

## Results

### Blood glucose after injection of streptozotocin

Rats were made diabetic with streptozotocin. Diabetes was confirmed by measuring the level of plasma glucose. They were designated as non-diabetic rats (<14 mmol/l plasma glucose) and diabetic rats (>14 mmol/l plasma glucose).

About 13.9% (11/79 rats) of the rats responded to the streptozotocin injection (blood glucose>14 mmol/l) according to fasting blood glucose, and 74.7% (59/79) of the rats responded to the streptozotocin injection (blood glucose>14 mmol/l) according to random blood glucose. Based on the later data of the experiment, random blood glucose testing was more reliable for distinguishing diabetic rats from non-diabetic rats. Fifty-nine diabetic rats were then used throughout experiment. One month after the diabetic rat model was set, five rats had to be rejected because blood glucose had fallen below 14 mmol/l (one rat each from Group II, V, and VII and two rats from Group III).

### The change of rat's weight

There was no statistically significant difference in the weight gain among the diabetic rats (p>0.05; Table 1). The diabetic rats had lower weight gain compared to the normal rats during the experimental period (p<0.05). However, treatments of CA, AG, or ASA to diabetic rats did not normalize bodyweights to a significant extent (Group II−VI).

**Table 1:** The bodyweight of all groups of rats during the experiments.

**Time**	**Body weight (g)**						
	**Group I**	**Group II**	**Group III**	**Group IV**	**Group V**	**Group VI**	**Group VII**
Before injection	143± 16.17 (n=5)	120.4±13.57 (n=9)	116±26.7 (n=9)	112.2±19.9 (n=9)	116±18.48 (n=9)	119.8±11.95 (n=9)	112.9±18.9 (n=14)
1 wk	234.6±18.13 (n=5)	171.6±19.7 (n=8)	168.1±36.57 (n=8)	146.8±30.6 (n=9)	167±27.3 (n=9)	157.4±23.2 (n=9)	169±22.6 (n=13)
2 wk	283.2±17.46 (n=5)	189.1±27.70 (n=8)	184.6±40.59 (n=7)	164.2±34.2 (n=9)	187±27 (n=8)	174.9±28.1 (n=9)	191.6±22.6 (n=13)
4 wk	378.4±13.83 (n=5)	191.4±61.10 (n=8)	196.1±61.38 (n=7)	157.2±55.1 (n=9)	204.9±49.6 (n=8)	159.9±43.8 (n=9)	219.8±37.7 (n=13)
5 wk	429±15.39 (n=5)	222±64.17 (n=8)	231.7±69.5 (n=7)	171.1±57.5 (n=9)	224.3±55.4 (n=8)	183.6±48.3 (n=9)	227.4±52.2 (n=13)
6 wk	452±15.60 (n=5)	232.7±66.12 (n=8)	239.6±72.75 (n=7)	176.4±60.8 (n=9)	236.6±62.4 (n=8)	188.1±50.2 (n=9)	245.4±52.9 (n=13)
7 wk	488.6±14.28 (n=5)	225.2±77.92 (n=8)	240.8±83.1 (n=7)	172.9±74.5 (n=9)	235.4±75.3 (n=8)	171.6±59.6 (n=9)	238.7±68.7 (n=13)
8 wk	498±22.90 (n=5)	233.7±87.03 (n=8)	247.4±90.37 (n=7)	173.3±71.9 (n=9)	248.9±71.9 (n=8)	184.1±66.0 (n=8)	248.6±73.5 (n=13)
9 wk	532.6±22.31 (n=5)	237.4±109.1 (n=8)	254.8±99.0 (n=7)	170.7±77.2 (n=9)	247.6±88.6 (n=7)	181.1±58.3 (n=7)	236.5±90.8 (n=13)
10 wk	542.8±17.74 (n=5)	233.8±104.2 (n=8)	249±104.3 (n=7)	183.0±73.9 (n=8)	246.5±93.3 (n=7)	168.6±60.0 (n=7)	238.5±86.1 (n=13)
11 wk	558.4±27.79 (n=5)	247.5±105.4 (n=7)	265.3±102.3 (n=6)	173.4±74.6 (n=8)	242.3±100.1 (n=7)	170.2±74.7 (n=6)	257.6±83.7 (n=10)
12 wk	550±34.46 (n=5)	244.8±112.7 (n=7)	265.4±114.1 (n=5)	159.9±67.9 (n=8)	252.3±107.9 (n=7)	143.3±54.0 (n=6)	246.8±89.9 (n=10)
13 wk	550.4±37.70 (n=5)	242.1±117.7 (n=7)	289.3±104.7 (n=5)	167.3±58.8 (n=7)	248.1±105.0 (n=7)	142.6±55.2 (n=5)	244.5±89.5 (n=10)

The data are the mean±SD. Weights are significantly different between Group I and Group II-VII after STZ injection. There are no significant differences between the treated diabetic rats (from Group II-VI) and the untreated diabetic rats (Group VII) two weeks after STZ injection. However, groups IV and VI were significantly lighter than the untreated diabetic rats from week 4 through week 13 (p<0.05). On the eighth week of diabetes, blood glucose of rat number in group V was 13.3 mmol/l (<14 mmol/l), and the rat died. Then, on the eighth week of diabetes, one rat was lost. Finally, five rats were taken out because of low blood glucose, and 14 rats were lost because of death. One rat is an overlap. The total number of rats studied was then 18. Group I: Normal; Group II: Carnosine treated (5 mg/ml); Group III: Carnosine treated (10 mg/ml); Group IV: Aspirin treated; Group V: Aminoguanidine treated (1 mg/ml); Group VI: Aminoguanidine treated (2 mg/ml); and Group VII: Untreated diabetic.

### Change in blood glucose

The diabetic rats have a higher blood glucose level compared to the normal rats during the experimental period (Table 2). There was no statistically significant difference in the blood glucose levels between the different groups of diabetic rats (p>0.05). Glucose levels remained stable throughout the experiment.

**Table 2:** Bodyweights of all groups of rats during the experiments.

**Time**	**Blood glucose (mmol/l)**						
	**Group I**	**Group II**	**Group III**	**Group IV**	**Group V**	**Group VI**	**Group VII**
72 h	6.8±0.6 (n=5)	29.2±6.1 (n=9)	30.5±6.3 (n=9)	31.0±2.9 (n=9)	28.0±6.5 (n=9)	32.6±1.0 (n=9)	29.8±4.2 (n=14)
4 wk	5.8±0.3 (n=5)	29.7±8.6 (n=8)	28.2±8.7 (n=7)	31.7±2.5 (n=9)	28.49±7.8 (n=8)	33.0±0.5 (n=9)	27.0±8.1 (n=13)
8 wk	6.2±0.6 (n=5)	29.3±8.4 (n=8)	27.5±11.6 (n=7)	29.2±3.8 (n=9)	27.3±9.3 (n=8)	30.1±2.6 (n=8)	28.2±5.7 (n=13)
12 wk	5.78±1.1 (n=5)	29.2±9.0 (n=7)	25.1±13.1 (n=5)	30.7±3.7 (n=8)	26.7±9.7 (n=7)	30.0±3.4 (n=6)	28.7±6.3 (n=10)
13 wk	5.9±0.9 (n=5)	29.5±9.4 (n=7)	24.8±13.4 (n=5)	32.4±1.6 (n=7)	27.7±10.2 (n=7)	29.6±5.2 (n=5)	28.5±6.8 (n=10)

The data are the mean±SD. Blood glucose levels in all groups of diabetic rats (Group II−VII) were significantly higher than in the normal rats (Group I). There were no significant differences between the six diabetic groups at any given stage (Group II−VII). Group I: Normal; Group II: Carnosine treated (5 mg/ml); Group III: Carnosine treated (10 mg/ml); Group IV: Aspirin treated; Group V: Aminoguanidine treated (1 mg/ml); Group VI: Aminoguanidine treated (2 mg/ml); and Group VII: Untreated diabetic.

### Corneal staining following eye drop use

The extent of corneal stain was observed throughout the experiment. There was no significant staining in the cornea from normal rats. With the progress of diabetes, fluorescent staining on the cornea gradually appeared in diabetic rats. Compared with normal rats, corneal staining points (median) increased slowly four weeks after STZ injection (Table 3). Four weeks after STZ injection there were significant differences between normal rats and all groups of diabetic rats (p<0.05), and corneal staining increased with the period of diabetes, indicating the defect of the cornea occurred in diabetes. However, no significant differences between groups of diabetic rats were noted at 4, 8, and 12 weeks after STZ injection (p>0.05), confirming the safety of eye drops used in the current study. In addition, no corneal opacity was observed in diabetic rats, but minor corneal ulcers appeared in severely diabetic rats.

**Table 3:** Corneal staining of all rats in different periods.

**Time**	**Corneal staining**						
	**Group I**	**Group II**	**Group III**	**Group IV**	**Group V**	**Group VI**	**Group VII**
72 h	0 (n=5)	0 (n=9)	0 (n=9)	0 (n=9)	0 (n=9)	0 (n=9)	0 (n=14)
4 wk	0 (n=5)*	1 (n=8)	0+ (n=7)	1 (n=9)	1 (n=8)	1+ (n=9)	1 (n=13)
8 wk	0 (n=5)*	1+ (n=8)	1 (n=7)	1+ (n=9)	2+ (n=8)	2+ (n=8)	2 (n=13)
12 wk	0 (n=5)*	2+ (n=7)	2 (n=5)	3 (n=8)	3 (n=7)	3 (n=6)	3 (n=10)

Values are expressed as medians. The “+” represents the middle of that median. The extent of corneal staining was graded according to the quantity of corneal staining: Grade 0: no staining; grade 1: up to a quarter of the cornea stained; grade 2: a quarter to half of the cornea stained; grade 3: more than half of cornea stained. The asterisk indicates that p<0.05 (Group I compared with the diabetic rats). There were no significant differences between any of the diabetic rats (Group II−VII) at any given stage. Group I: Normal; Group II: Carnosine treated (5 mg/ml); Group III: Carnosine treated (10 mg/ml); Group IV: Aspirin treated; Group V: Aminoguanidine treated (1 mg/ml); Group VI: Aminoguanidine treated (2 mg/ml); and Group VII: Untreated diabetic.

### Progression of lens opacification

**Figure 2:**
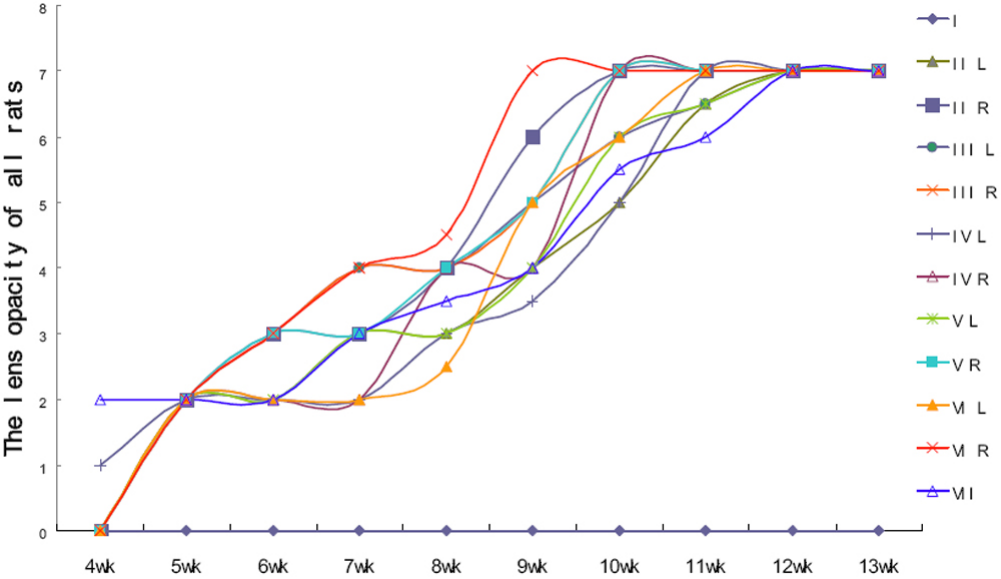
Lens opacity of all groups on difference experimental periods. All of the diabetic rats developed cataracts more or less at the same rate. The lens progressed rapidly to opacity from the 6th week to 10th weeks after STZ injection. Values are expressed as medians. The “+” represents the middle of that median. Median representation: 0, clear; 1, clear nucleus with wide sutures; 2, slight dense nucleus with opacities radiating from sutures; 3, dense nucleus without clefts; 4, dense nucleus with clefts; 5, nuclear cataract with clefts; 6, nuclear cataract with dense radial opacities; 7, nuclear cataract with whole lens opacity.

All of the diabetic rats developed cataracts more or less at the same rate. The lens did not progress to opacity until the fourth week after STZ injection (Table 4, Figure 2). Unlike the diabetic rats, the control rats retained clear lenses throughout. The untreated diabetic rats seemed to progress to grade 2 cataract faster than the treated groups (Table 4, Figure 2). After five weeks, all diabetic groups had reached grade 2. After that, there seemed to be little difference between the diabetic groups. However, the progression of cataracts in all treated groups was slightly delayed between the sixth week and eighth week. The difference between groups was not statistically significant at this time.

**Table 4:** Lens opacity of all groups at different experimental periods.

**Time**	**Lens opacity**											
	**Group I**	**Group II**		**Group III**		**Group IV**		**Group V**		**Group VI**		**Group VII**
		**L**	**R**	**L**	**R**	**L**	**R**	**L**	**R**	**L**	**R**	
4 wk	0 (n=5)	0^a^	0 (n=8)	0^b^	0 (n=7)	1	0 (n=9)	0	0 (n=8)	0	0 (n=9)	2 (n=13)
5 wk	0 (n=5)	2	2 (n=8)	2	2 (n=7)	2	2 (n=9)	2	2 (n=8)	2	2 (n=9)	2 (n=13)
6 wk	0 (n=5)	2	3^c ^(n=8)	3	3 (n=7)	2	2 (n=9)	2	3^e^ (n=8)	2	3 (n=9)	2 (n=13)
7 wk	0 (n=5)	3	3 (n=8)	4	4 (n=7)	2	2 (n=9)	3	3 (n=8)	2	4 (n=9)	3 (n=13)
8 wk	0 (n=5)	3	4 (n=8)	4	4 (n=7)	3	4 (n=9)	3	4 (n=8)	2+	4+^f ^(n=8)	3+ (n=13)
9 wk	0 (n=5)	4	6^c^ (n=8)	5	5 (n=7)	3+	4 (n=9)	4	5^e ^(n=7)	5	7 (n=7)	4 (n=13)
10 wk	0 (n=5)	5	7^c^ (n=8)	6	7^d^ (n=7)	5	7 (n=8)	6	7^e^ (n=7)	6	7 (n=7)	5+ (n=13)
11 wk	0 (n=5)	6+	7 (n=7)	6+	7 (n=6)	7	7 (n=8)	6+	7 (n=7)	7	7 (n=6)	6 (n=10)
12 wk	0 (n=5)	7	7 (n=7)	7	7 (n=5)	7	7 (n=8)	7	7 (n=7)	7	7 (n=6)	7 (n=10)
13 wk	0 (n=5)	7	7 (n=7)	7	7 (n=5)	7	7 (n=7)	7	7 (n=7)	7	7 (n=5)	7 (n=10)

Values are expressed as medians. The “+” represents the middle of that median. Median representation: 0, clear; 1, clear nucleus with wide sutures; 2, slight dense nucleus with opacities radiating from sutures; 3, dense nucleus without clefts; 4, dense nucleus with clefts; 5, nuclear cataract with clefts; 6, nuclear cataract with dense radial opacities; and 7, nuclear cataract with whole lens opacity. Note: ^a^p=0.014 (group II compared with group VII on the fourth week); ^b^p=0.043 (group III compared with group VII on the fourth week); ^c^p<0.05 (the left eyes compared with the right ones in the lens opacity at week 6, 9, and 10 in group II); ^d^p<0.05 (the left eyes compared with the right ones in the lens opacity at week 10 week in group III); ^e^p<0.05 (the left eyes compared with the right ones in the lens opacity at week 6, 9, and 10 in group V); and ^f^p<0.05 (the left eyes compared with the right ones in the lens opacity on the eighth week in group VI). L: the left eyes, which served as the treated eyes; R: the right eyes, which served as the control eyes. The number of lens (n) represents equally in both the left and right eyes in each group. Group I: Normal; Group II: Carnosine treated (5 mg/ml); Group III: Carnosine treated (10 mg/ml); Group IV: Aspirin treated; Group V: Aminoguanidine treated (1 mg/ml); Group VI: Aminoguanidine treated (2 mg/ml); and Group VII: Untreated diabetic.

CA treatment delayed the progression of cataracts in diabetic rats, and the delay was statistically significant on the fourth week of diabetes (p<0.05, when compared with the untreated diabetic rats). However, opacification progressed after that and the difference was lost after five weeks. AG seemed to protect for 11−13 weeks, but there were only five surviving rats in the low aminoguanidine treatment group during this period. Therefore, this may result from the random error due to low numbers for analysis. ASA treatment had no effect on the progression of lens opacities at any time (p>0.05, when compared with the untreated diabetic rats).

In comparison with the left eyes (treated) and right eyes (control), there were significant differences in lens opacity between the left eyes and the right ones on week 6, 9, and 10 of diabetes in groups II and V. The same statistical difference was also observed on week 10 in group III and week 8 in group VI. (p<0.05; Table 4, Figure 2), which indicates less opacity in the treated eyes than the untreated eyes even in the same rat.

### Activity of enzymes

The antioxidant enzymes, catalase (CAT) and the reducing peptide glutathione (GSH), decreased in lenses of untreated diabetic rats 13 weeks after the STZ injection (Table 5; p<0.05, when compared with the control rats−group I). CA had a statistically significant effect to raise the activity of CAT (groups II and III). ASA seemed to raise CAT levels above control (group I), but the differences were not statistically significant (p>0.05; Table 5). However, AG seemed to decrease CAT levels below the control (group I), suggesting that either AG inhibits CAT or it interferes in the assay.

**Table 5:** Activity of several enzymes and the levels of proteins in the left lens of rats 13 weeks after injection.

**Time**	**Group I (n=5)**	**Group II (n=7)**	**Group III (n=5)**	**Group IV (n=7)**	**Group V (n=7)**	**Group VI (n=5)**	**Group VII (n=10)**
CAT	34.0±21.2	96.6±62.5^a^	111.0±69.0^b^	94.9±21.2	22.6±18.2	14.0±15.2	28.7±20.3*
GR	5.2±2.7	7.6±3.9^c,e^	10.4±3.1^d,e^	5.6±5.0	5.5±3.6	4.1±3.7	5.5±4.3
GSH	102.7±39	76.3±19.5	41.4±13.8	42.5±22.1	66.3±26.9	75.7±45.2	55.2±32.6**
Lens water-soluble proteins	4.9±0.4	4.4±0.5^f^	4.32±0.9^f^	4.4±0.7^f^	4.3±0.5^f^	4.4±0.5^f^	4.7±0.4**

The data are the mean±SD. CAT (catalase) is expressed as µmol/g lens protein. GSH (glutathione) is expressed as mg/g lens protein. GR (glutathione reductase) is expressed as µmol/g lens protein. The water-soluble protein is expressed as g/l. An asterisk indicates that p<0.05 (group I compared with group VII). A double asterisk indicates that p<0.001 (group I compared with group VII). Note: ^a^p<0. 001 (group II compared with group VII); ^b^p=0.002 (group III compared with group VII); ^c^p=0.003 (group II compared with group VII); ^d^p<0.001 (group III compared with group VII); ^e^p<0.05 (groups II-III compared with group I); ^f^p<0. 001 (group II-VI compared with group I). There were no significant differences in the lens water-soluble proteins in any of the six diabetic groups (Group II-VII). Group I: Normal; Group II: Carnosine treated (5 mg/ml); Group III: Carnosine treated (10 mg/ml); Group IV: Aspirin treated; Group V: Aminoguanidine treated (1 mg/ml); Group VI: Aminoguanidine treated (2 mg/ml); and Group VII: Untreated diabetic.

Glutathione reductase (GR) did not decrease in the lens of the untreated diabetic rats 13 weeks after injection (Table 5) whereas CA seemed to have raised GR activity (p<0.05, when group I was compared with group VII. The water-soluble proteins decreased in the untreated diabetic rats (Table 5). The three treatments did not significantly prevent these declines.

There were hyperglycemia-dependent increases in the glycated protein (Table 6). Significant differences in the glycated protein between the control (Group I) and the diabetic groups (Group II−VII) were noted. The three drops treatments had an effect in decreasing glycated protein formation (groups II−IV compared with group VII). No differences between the left eyes (treated) and right eyes (control) were observed, suggesting that the eyes drops on one eye may have effects on the other side through the blood-ocular barrier.

**Table 6:** Levels of glycated proteins in the left and right lens of rats 13 weeks after injection.

		**Group I (n=5)**	**Group II (n=7)**	**Group III (n=5)**	**Group IV (n=7)**	**Group V** **(n=7)**	**Group VI (n=5)**	**Group VII (n=10)**
Glycated proteins	L	4.2±0.4	4.0±1.2**	3.4±0.3**	3.7±0.5**	4.5±0.7**	4.1±0.3**	5.4±0.5*
	R	4.2±0.4	4.1±0.5**	3.6±0.6**	3.5±0.3**	4.9±0.8**	4.3±0.5**	5.4±0.5*

The data are the mean±SD. Glycated lens protein is expressed as mmoles HMF/mole protein. An asterisk indicates that p<0.001 when group I is compared with group VII, and a double asterisk indicates that p<0.001 for groups II-VI when compared with group VII. There were no significant differences between the left and right lens in any of the five treated diabetic groups. L: the left eyes that served as the treated eyes; R: the right eyes that served as the control eyes. The number of lens (n) represents equally in both the left and right eyes in each group. Group I: Normal; Group II: Carnosine treated (5 mg/ml); Group III: Carnosine treated (10 mg/ml); Group IV: Aspirin treated; Group V: Aminoguanidine treated (1 mg/ml); Group VI: Aminoguanidine treated (2 mg/ml); and Group VII: Untreated diabetic.

## Discussion

In the present study, three putative anti-cataract drugs, CA, AG, and ASA, administered as eye drops, were investigated in parallel to assess which can delay diabetic cataract in rats. The three types of eye drops were used for the first time in a streptozotocin-induced cataract study and showed an inhibition of cataract at an early stage. CA eye drops produced a better inhibitory effect than the other two drugs.

Numerous laboratory studies suggest that cA has antiglycating, antioxidant, and free-radical scavenging activity [7,11-13]. Its potential benefit against cataract formation was reported [14,20,22]. Our data showed that CAT enzyme activity was significantly decreased in the untreated diabetic rats (group VII) but less so in the CA-treated diabetic rats. CA raised CAT and GR levels above the control (group I), suggesting that the part of the mechanism of the protective effect of carnosine against diabetic cataract is by protecting catalase and GR, which in turn generally protect against peroxide and oxidation.

Evidence from epidemiological, in vitro, and animal studies has accumulated to support the idea that ASA-like drugs protect against cataract. ASA delivered orally can delay diabetic cataract in rats [31], topical ASA can provide protection against galactosemic cataract [38] and protect against dexamethasone-induced cataract in cultured rat lens [34]. However, there was no significant preventive effect on diabetic cataract in this study by using ASA drops (Group IV). This may be due to the severity of the diabetes. More loss of bodyweight was observed in Group IV, although the blood glucose in the diabetic groups was all above 30 mmol/l. The presented results indicated that the activity of CAT was increased similarly in the ASA-treated diabetic rats. However, there was no statistically significant difference when compared with the untreated diabetic rats. Thus, ASA at such dosage did not have enough of an effect in inhibiting oxidation unless the level of blood glucose was not relatively too high for ASA against diabetic cataract.

AG can inhibit a variety of diabetic complications, including cataract [23,26,28,29]. The current data showed that there was no significant prevention by AG eye drops on diabetic cataract. Compared with the non-treated diabetic group (Group VII), the high dose AG-treated group (Group VI) had greater bodyweight loss, suggesting the serious diabetic effect in this group. All measurements of blood glucose were above 30 mmol/l in these diabetic rats, which may have decreased the chances of protection. Moreover, CAT activity was significantly decreased in the AG-treated diabetic rats when compared with the untreated diabetic rats (Table 5). This indicates that protection against peroxide may not contribute to the protective effect of AG against diabetic cataract, especially at our high levels of hyperglycemia.

Lens opacification is a complex phenomenon. Glycation is one of the contributory factors in lens opacification in this animal model. The present results showed that the level of lens protein glycation in the untreated diabetic rats was significantly higher than that in the normal rats. But diabetic rats treated with CA, ASA, and AG had a lower level of glycated protein compared to the untreated diabetic rats, suggesting that CA, ASA, and AG inhibited the formation of glycated protein. If glycation was the only factor, normalization of glycation levels seen in treated diabetic rats should have decreased lens opacification. However, there was only a delay in these groups, suggesting that other factors also play some role. Interestingly, we also observed that they inhibited glycation on the right eyes although applied only to the left eyes. This indicates that the drops may function through blood-ocular barrier. The unique anatomy and physiology of the eye offers many challenges to developing effective drug delivery systems. Historically, drugs have been administered to the eye as liquid drops instilled in the cul-de-sac. The transport of molecules among the eye to the other eye or vitreous/retina and systemic circulation is restricted by the blood–retinal barrier [39]. The present observation showed that glycation can be inhibited by these small molecules through the blood-ocular barrier. However, the function still remains to be developed.

We conclude that CA, ASA, and AG can inhibit glycation and thus the formation of AGEs. CA seems to be effective in delaying the progression of lens opacification in diabetic rats only during the earlier stages compared with AG and ASA and may be associated with the protective effect against the inactivation of enzymes and the formation of AGEs. Unfortunately, enzymes and GSH could be measured only after 13 weeks, the end of the experiment, whereas the benefit of CA was noted at four weeks. Further study of CA and other potential drugs on their biological features and their mechanisms of delaying the progression of cataractogenesis may provide a new therapy for preventing cataract.
